# Emerging trends and research hotspots in atopic dermatitis-related itch over the past 10 years: a bibliometric and visual analysis

**DOI:** 10.3389/fmed.2025.1503312

**Published:** 2025-06-17

**Authors:** Weiyu Wu, Fubing Yu, Xiaoli Tao, Xiongbo Yang, Xiaoling Xue, Bangtao Chen

**Affiliations:** ^1^Department of Dermatology, Chongqing University Three Gorges Hospital, School of Medicine, Chongqing University, Chongqing, China; ^2^Department of Dermatology, The Third Affiliated Hospital of Chongqing Medical University, Chongqing, China; ^3^Department of Pathogenic Biology, College of Basic Medical Sciences, Jinzhou Medical University, Jinzhou, China

**Keywords:** bibliometric analysis, itch, mechanism, neuron, dupilumab, atopic dermatitis

## Abstract

**Introduction:**

Atopic dermatitis (AD) is a chronic inflammatory skin disorder that causes itching. While numerous studies on AD-related itch exist, a bibliometric analysis has not been performed on this topic. The study aimed to investigate research trends and hotspots in the field of AD-related itch over the last decade.

**Methods:**

We retrieved publications relevant to AD-related itch published from 2014 to 2023, within the Science Citation Index-Expanded of Web of Science Core Collection. We conducted a bibliometric and visual analysis involving annual publications, nations, institutions, journals, authors, co-citation references, and keywords by utilizing VOSviewer, CiteSpace, and the bibliometric online analysis platform.

**Results and discussion:**

A total of 2534 articles and reviews were retrieved. Overall, the number of publications on AD-related itch has risen steadily over the past decade. The USA was found to be the most influential nation. Simpson et al. were found to be the most prolific author. Over the past 10 years, research has primarily revolved around barrier function, itching mechanism, sensory neurons, and therapeutic drugs for AD. The mechanisms of AD-related itch and advanced drugs are the current research trends. This study can serve as a foundation for further research.

## 1 Introduction

Atopic dermatitis (AD) is a common chronic inflammatory skin disorder that affects patients’ quality of life, learning efficiency, work productivity, and even mental health. The most disruptive symptom of AD is itching. Itching is triggered by various irritants and is transmitted via cutaneous sensory nerve fibers to the spinal cord through afferent fibers. Itching can damage the skin barrier via the itch–scratch cycle, enabling allergens and irritants to penetrate the skin and exacerbating inflammatory signals. The progress of itch neuroimmunity reveals that AD-related itch signals can be encoded by sensory neurons, which can be activated directly by multiple immune cells and cytokines (1). However, the pathophysiological mechanism of AD-related itch remains unclear, complicating the treatment of AD-related itch.

A bibliometric analysis examines the characteristics of publications over a specific period. To the best of our knowledge, no bibliometric analysis focusing on AD-related itch has been published. Hence, we conducted this study to evaluate the current situation and hotspots in this field in the period spanning from 2014 to 2023, potentially serving as a basis for further research.

## 2 Materials and methods

### 2.1 Data sources and search strategies

We carried out a bibliometric analysis using the Science Citation Index Expanded (SCIE). The search terms and strategy were as follows: (TS = (“atopic dermatitis”) OR TS = (“atopic eczema”)) AND (TS = (itch) OR TS = (Pruritus)) AND (Language = (English)), within a limited time frame, from 2014 to 2023. Among the publications, only articles and review articles were considered. The details are provided in Supplementary Table 1.

### 2.2 Data collection

We extracted data such as titles, keywords, nations, authors, institutions, journals, and the number of citations from the screened publications. Subsequently, the data was converted to TXT format and imported into the data into the Online Analysis Platform of Bibliometry,^1^ VOSviewer v1.6.10.0, and CiteSpace (version 5.8. R3) for further analysis.

### 2.3 Bibliometric analysis

We performed a bibliometric analysis of various literature characteristics, including countries, institutions, authors, journals, references, and keywords. The number of publications (NP) was used as a measure of productivity, while the number of citations (NC) excluding self-citations served as an indicator of impact. The average citations per year (ACY) score was adopted to eliminate time bias. The H-index was used to determine a researcher’s academic contribution (2). The Impact Factor (IF) is a widely acknowledged measure for evaluating the quality and impact of medical journals (3). The Global Citation Score (GCS) is a crucial measure of an article’s contribution (4). We generated bibliometric maps using VOSviewer to obtain comprehensive information through the analysis of co-citation and co-occurrence (5). In CiteSpace, we employed cluster analysis, timeline, references, and keyword bursts to visually assess the current situation and identify emerging trends in the domain (4).

## 3 Results

### 3.1 Quantity and trends analysis of publications

**Figure 1:**
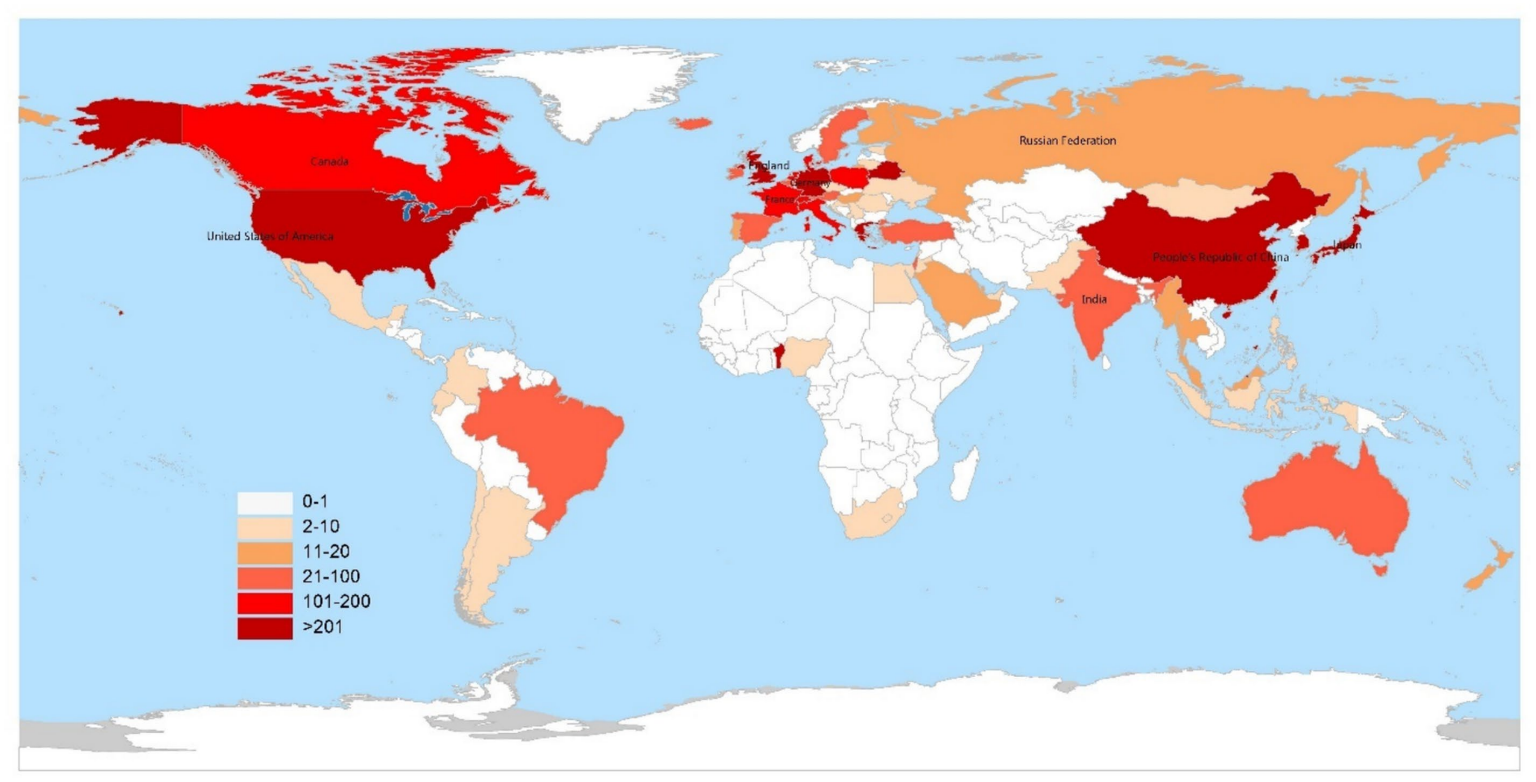
Geographical distribution of publications on atopic dermatitis-related itch, 2014–2023.

**Figure 2:**
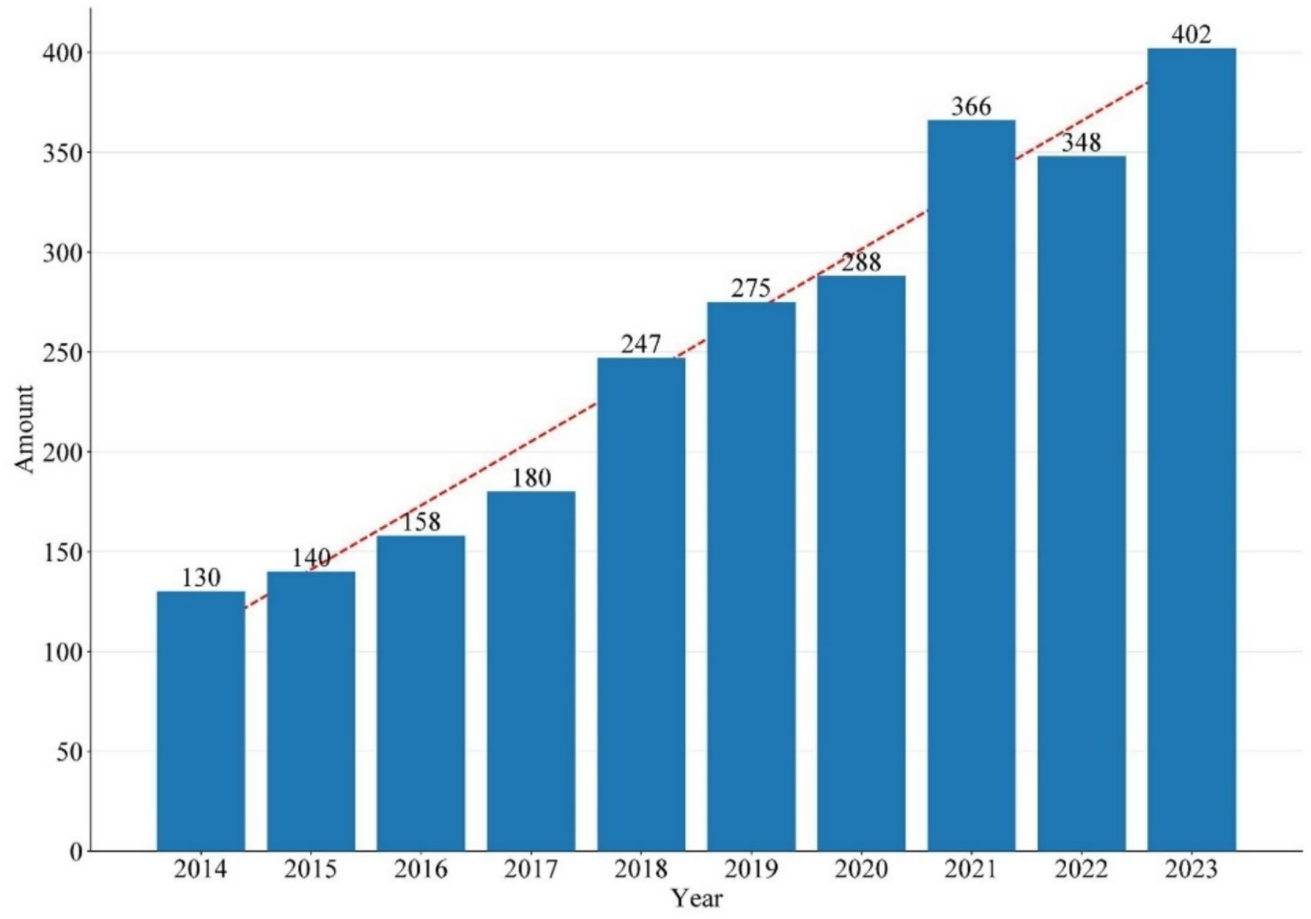
Number of publications by year during the last 10 years.

**Figure 3:**
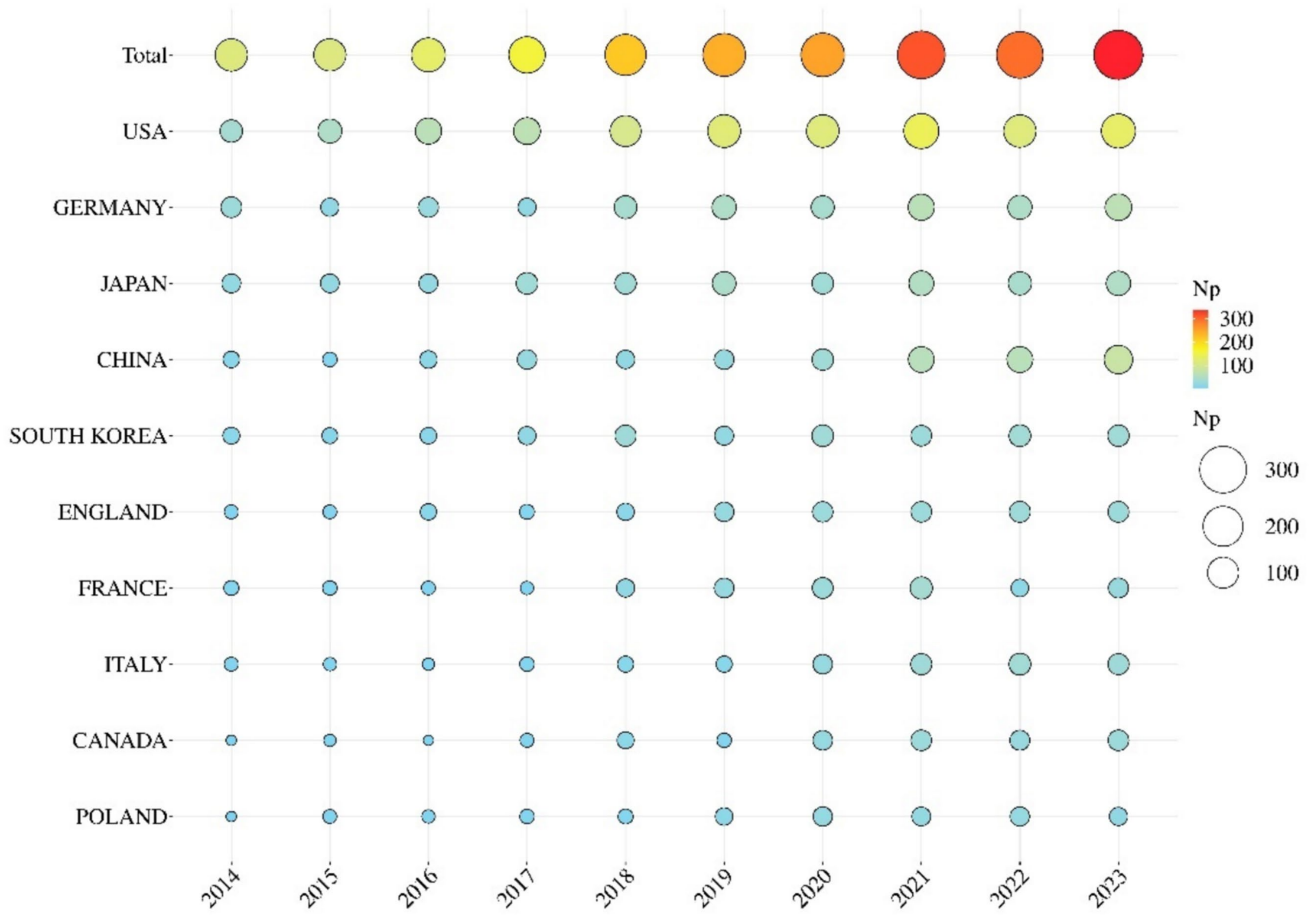
Top 10 countries in terms of annual publications on AD-related itch research, from 2014 to 2023.

In total, 2,534 articles published from 2014 to 2023 were retrieved from the Science Citation Index Expanded (SCI-E) of the Web of Science Core Collection (WoSCC). Figure 1 shows the geographical distribution of publications on AD-related itch, with most contributions originating from countries in Europe, America, and Asia. Figure 2 shows the number of annual publications, which increased from 130 in 2014 to 402 in 2023. A polynomial-fitting curve depicts the yearly trend in publication volume. In general, the increase in the NP indicates that AD-related itch research has experienced rapid development recently. The top 10 countries were ranked by NP and are presented in Supplementary Table 2. The country that published the majority of papers was the United States, followed by Germany, Japan, China, and South Korea. The United States had the highest number of publications, with 924 publications, a total of 29,248 citations, and the highest H-index (88). Germany had the highest ACY (48.6). In Figure 3, the size and hues of the circle symbolize the volume of the papers. The larger the volume of papers published, the larger the circle and the hues shift from light blue to red. The NP in China showed a significant increase, and the United States contributed the most to this research field.

### 3.2 Analysis of affiliation

Supplementary Table 3 presents the top 10 most productive affiliations. The top seven affiliations were all from the United States. Among them, Northwestern University had the highest NP (148). The Feinberg School of Medicine had the highest NC (16296), H-index (50), and ACY (275.37).

### 3.3 Analysis of authors

Supplementary Table 4 lists the top 10 most productive authors. They were primarily from the United States, Germany, Japan, and Denmark. They authored 541 publications, which constituted 21.3% of the overall number of papers. Simpson, EL from Oregon Health & Science University had the highest NP (92), NC (6738), and H-index (37).

### 3.4 Analysis of journals

We identified the top 10 journals with the highest number of publications, as listed in Supplementary Table 5. Acta Dermato-Venereologica had the highest NP (93). The Journal of Allergy and Clinical Immunology recorded the highest NC (3975), and the Journal of the American Academy of Dermatology had the highest H-index (32).

### 3.5 Analysis of highly cited articles

**Figure 4:**
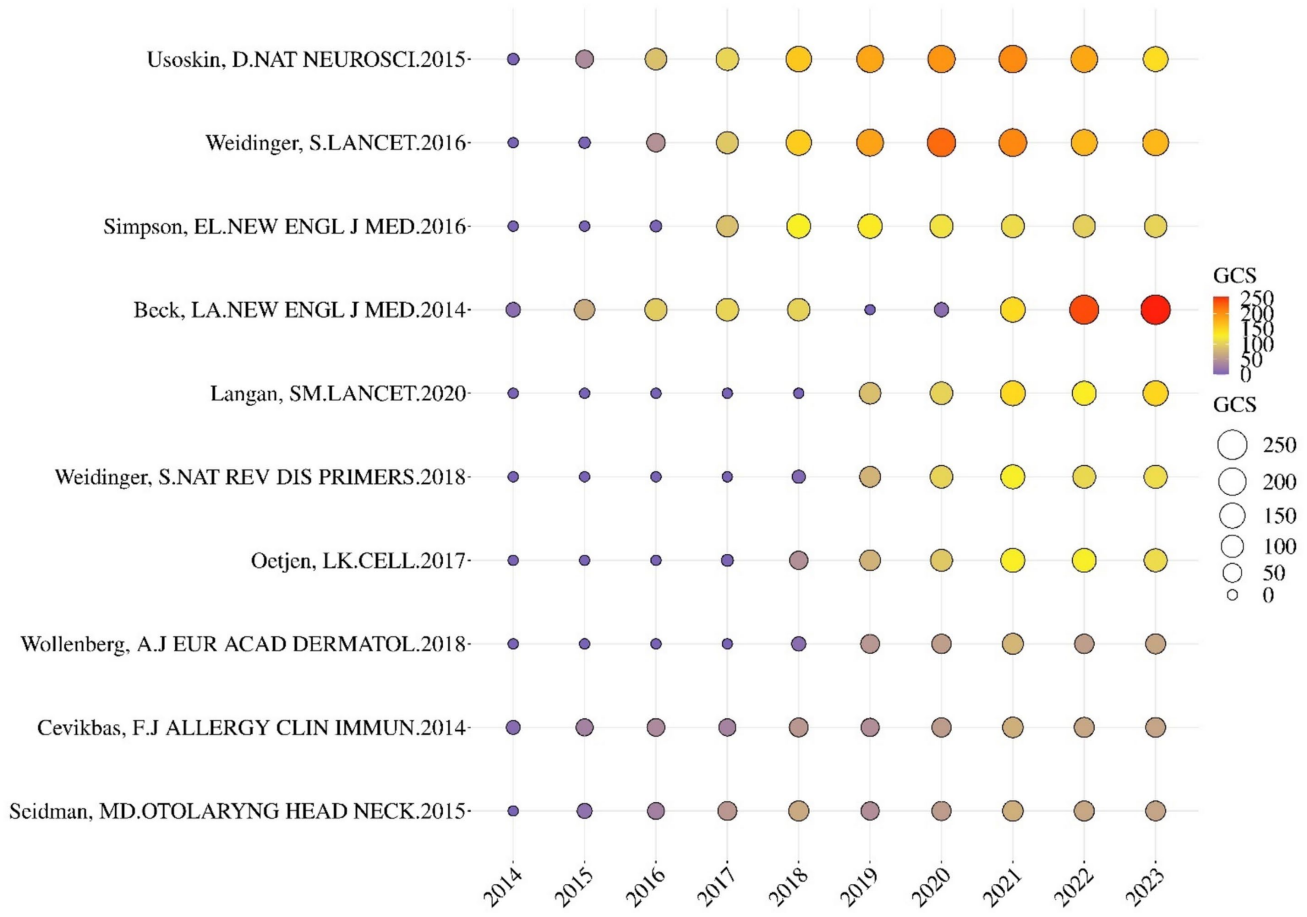
GCS’s annual number for the top 10 most cited articles.

Figure 4 shows the GCS’s annual number for the top 10 most cited articles. The size and hues of the circle indicate the GCS of the papers. The article titled *Unbiased Classification of Sensory Neuron Types by Large-scale single-cell RNA Sequencing* was cited the most, followed by *Atopic Dermatitis* and *Dupilumab Treatment in Adults with Moderate-to-Severe Atopic Dermatitis*. The third article had a marked increase in GCS, especially during 2022–2023. This finding indicates the significant influence this article has had in recent years.

### 3.6 Analysis of the research trend network

**Figure 5:**
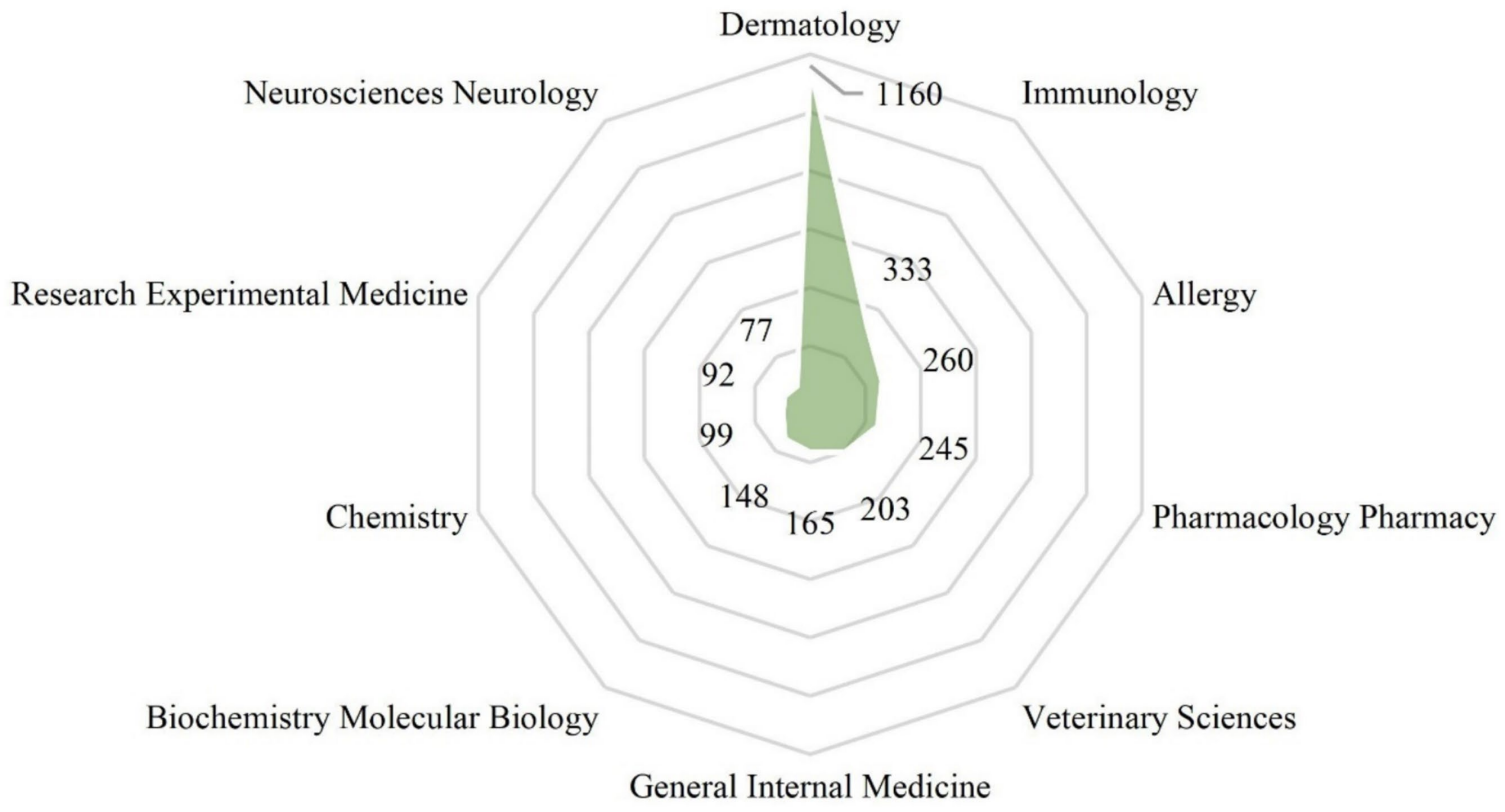
Top 10 productive categories of AD-related itch.

Figure 5 shows the top 10 productive categories of AD-related itch. Dermatology emerged as the most prevalent research area with 1,160 papers, followed by immunology (333 papers), allergy (260 papers), and pharmacology (245 papers). The aforementioned research themes have been identified as research trends over the past decade.

### 3.7 Analysis of co-citation and clustered

**Figure 6:**
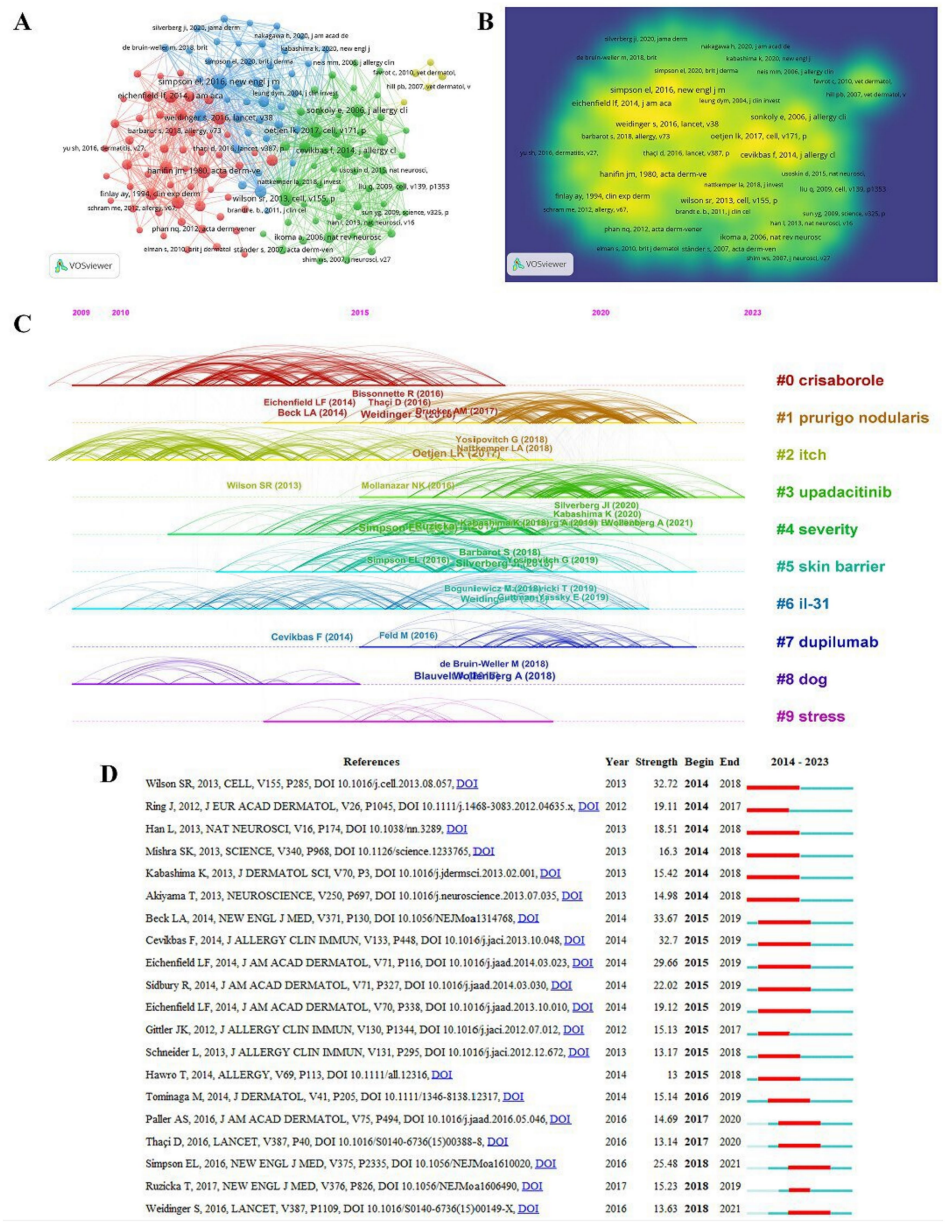
Network of co-citation. 154 references were chosen for co-citation analysis **(A)**. A map of density visualization for co-citation **(B)**. Timeline view for the top 10 clusters of co-citation **(C)**. The top 20 references with the strongest citation bursts **(D)**.

The networks of co-citation emphasize research topics in this specific field. We analyzed 2,534 publications and 59,994 references with CiteSpace software. We conducted a clustering analysis to ensure the homogeneity of highly cited literature on AD-related itch. Figure 6A provides a map of co-citation networks. Each node symbolizes a single article, with its size directly proportional to the frequency of citation. The connection between nodes indicates that they were cited in the same literature. The thickness of the lines indicates the degree of relevance between citations. The shorter the line, the stronger the association between the two citations. In addition, nodes with different colors were used to segregate the articles into distinct clusters. Of the 59,994 references, 154 (classified into 4 clusters) had been cited at least 50 times. Cluster 1 (in red) references primarily encompass the pathogenesis, epidemiology, clinical manifestations, methods for diagnosis and assessment, and treatment methods of AD (6). Cluster 2 (in green) references predominantly address the mechanism of AD, introducing novel mechanisms such as type 2 cytokines (7), interleukin (IL)-31, transient receptor potential vanilloid 1 (TRPV1), transient receptor potential cation channel subfamily A member 1 (TRPA1), and sensory neurons (8, 9). Cluster 3 (in blue) references addressed that dupilumab could significantly improve symptoms of AD (10) and quality of life (11). Cluster 4 (in yellow) focused on the diagnostic criteria that could be applied to dogs with AD (12). Figure 6B shows articles with a high frequency of co-citation in a map of density visualization. Articles in yellow areas are cited more frequently, whereas those in green are cited less frequently. The articles that were primarily published were collected from the following journals: The Lancet, New England Journal of Medicine, Journal of the American Academy of Dermatology Allergy, and Journal of Clinical Investigation. A timeline view of co-citation analysis for AD-related itch is shown in Figure 6C. The figure indicates the top 10 clusters. Each horizontal line represents a cluster, with the connections between the nodes denoting co-citation relationships. Timeline, positioned at the top, progresses from left to right, indicating the chronological development from earlier to more recent publications. The cluster label on the right presents the topic of the domain. The 10 clusters included #0crisaborole, #1 prurigo nodularis, #2 itch, #3 upadacitinib, #4 severity, #5 skin barrier, #6 interleukin (IL)-31, #7 dupilumab, #8 dog, and #9 stress. The citation burst of reference refers to an article exhibiting a sudden surge in citation frequency, which generally signals the emergence or a shift in the field. Furthermore, the reference with higher burst strength indicates greater significance within the field (13). Figure 6D displays the top 20 strongest citation bursts of references in AD-related itch research. The blue bars depict the publication periods of the references, while the red bars illustrate the periods of citation burst.

Among the burst references, the latest article was named Anti-IL-31 Receptor A Antibody for Atopic Dermatitis, published in The New England Journal of Medicine in 2017, and the burst occurred from 2018 to 2019. IL-31 secreted by T-helper type 2 cells is an influential pruritogenic cytokine (14). During the phase 2 trial, patients with moderate-to-severe AD who received monthly treatment with nemolizumab showed marked alleviation in the symptom of pruritus, thus demonstrating that nemolizumab effectively targets IL-31 receptor A in AD (15). The publication with the highest burst strength, which was published in the New England Journal of Medicine, reported that dupilumab could dramatically treat patients with AD without notable adverse reactions (5).

### 3.8 Analysis of the occurrence of keywords

**Figure 7:**
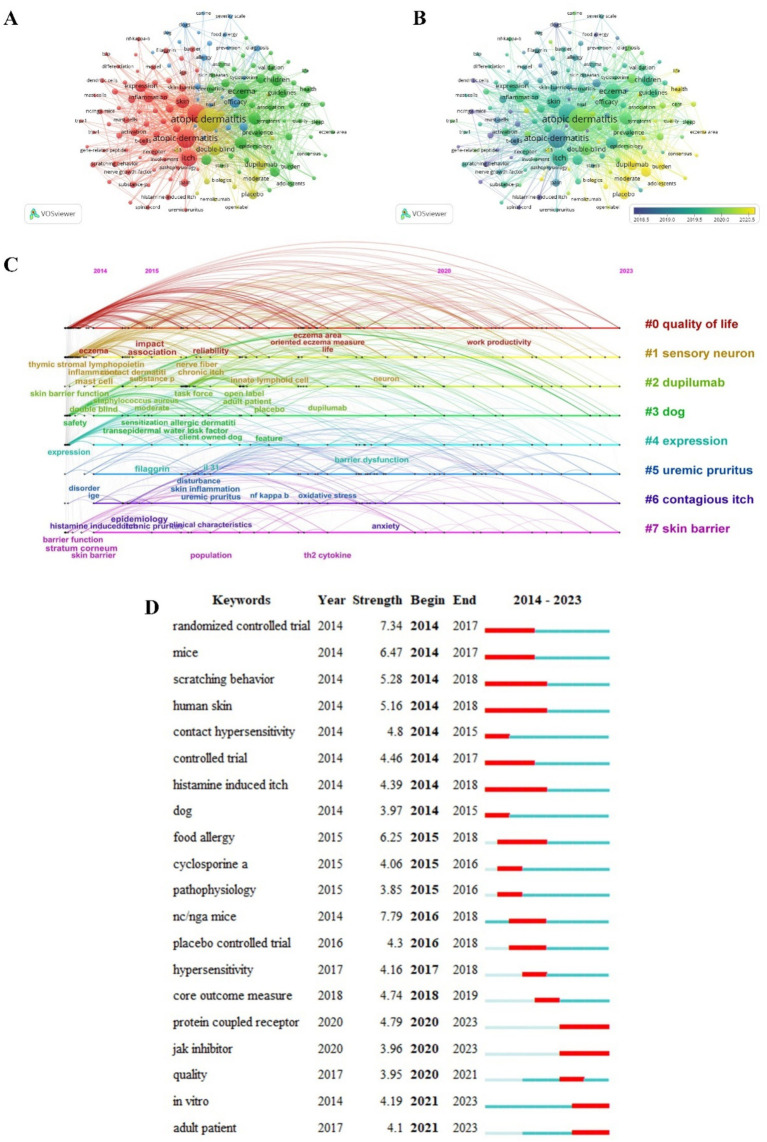
Network of keywords. 156 keywords were chosen for occurrence analysis **(A)**. A mapping of keyword overlay **(B)**. A clustering timeline of keyword co-occurrence **(C)**. The top 20 keywords with the most bursts **(D)**.

We analyzed 7,078 keywords collected from 2,534 publications. In Figure 7A, the node symbolizes the keyword, and the node’s size reflects the frequency of keyword occurrence. The lines connecting the nodes signify the co-occurrence of keywords. The closer the relationship between nodes, the thicker the line. There are 156 words that appeared at least 25 times and were categorized into 4 distinct clusters using VOSviewer. Cluster 1 (in red) is primarily concerned with the mechanism of AD-related itch. Cluster 2 (in green) focused on the epidemiology, burden, symptoms, and guidelines of eczema. Cluster 3 (in blue) is related to the complications of AD, including food allergies, asthma, and rhinitis. Cluster 4 (in yellow) focused on dupilumab, nemolizumab, and other biologics in AD treatment. Figure 7B maps the overlay of keywords. The varying colors represent the respective publication years, and keywords in yellow emerged later than those in purple. Figure 7C is a clustering timeline of the co-occurrence of keywords. The figure presents the top eight clusters. The connections between the nodes indicate the co-occurrence of keywords. The eight clusters included #0 quality of life, #1 sensory neuron, #2 dupilumab, #3 dog, #4 expression, #5 uremic pruritus, #6 contagious itch, and #7 skin barrier. Even across species, the incidence of AD is gradually increasing. Considerable research has been conducted on the prevalence, impact, and treatment of canine AD (16). Considering the similarities in mechanism between pruritus in AD and uremic pruritus, studies on uremic pruritus are gradually emerging (17). The analysis of keyword bursts as a valuable method could help discern trends in a field. Figure 7D shows the top 20 strongest keyword bursts on AD-related itch over the past decade. The keyword “Nc/Nga mice” had the strongest burst (7.79). The Nc/Nga mice are a suitable animal model, as they display clinical and histological similarities to human AD. The latest keywords were “protein-coupled receptor” and “Janus kinases (JAK) inhibitor” in 2020. “Protein-coupled receptor,” “jak inhibitor,” “*in vitro*,” and “adult patient” were hot keywords that appeared in recent years and could help scholars understand research hotspots. The map of density visualization for keywords co-occurrence showed that, except for the terms “atopic dermatitis” and “itch,” other high-frequency keywords included “expression,” “efficacy,” “dupilumab,” “double-blind,” “skin,” “inflammation,” and “children.”

## 4 Discussion

The study represents the first bibliometric analysis focused on AD-related itch, with 2,534 articles and reviews published between 2014 and 2023 analyzed. This study was used to identify core research areas from the past decade and to highlight emerging hotspots in this research field. As shown in Figures 1–3, the papers are mainly from the United States, Germany, Japan, and China. An increasing trend was observed in the studies being published, suggesting that a growing number of researchers are focusing on the field of AD-related itch (Supplementary Tables 3, 4). Among the top contributors, seven of the leading affiliations and five of the top 10 specialist scholars, based on NP, NC, H-index, and ACY, were from the United States, indicating that the United States has made the most significant contributions to research on AD-related itch. The Journal of Allergy and Clinical Immunology and the Journal of the American Academy of Dermatology were the most notable journals in terms of IF, H-index, and ACY (Supplementary Table 5). Analyzing journal sources can help scholars pinpoint key journals in a specific field.

The most influential cited articles included Unbiased Classification of Sensory Neuron Types by Large-scale Single-cell RNA Sequencing, published in Nature Neuroscience, and Dupilumab Treatment in Adults with Moderate-to-Severe Atopic Dermatitis, published in the New England Journal of Medicine (Figure 4). The first article revealed that itching induced by inflammatory skin diseases such as AD was associated with a distinct itch-generating type. The second article identified that dupilumab can significantly and rapidly improve symptoms of AD, regardless of the disease activity assessment.

The analysis of co-citations and keyword co-occurrence indicated that research in the early years concentrated on the mechanism of AD-related itch (Figures 6, 7). The itch mechanism involves damage to numerous aspects of the skin, including the skin barrier, deletion of the filaggrin gene, inflammation, IL-13, TRPA1/TRPV1, mast cells, and nerve growth factor (18). AD-related itch is triggered by diverse pruritogens that primarily consist of type 2 cytokines and histamine. Histamine, which is secreted by mast cells and basophils, combines with H1 and H4 receptors on histaminergic nerves and then activates TRPV1, which is crucial to inflammatory disease disorder (19). Type 2 cytokines, such as IL-4, IL-13, IL-31, and thymic stromal lymphopoietin (TSLP), prominently contribute to itching. TSLP expressed in cutaneous epithelial cells of patients with AD is predominantly released following mechanical trauma, such as scratching. TSLP produced by keratinocytes directly stimulates sensory neurons, thereby eliciting an itching sensation (20). The type-2 cytokines implicated in AD transmit signals via the JAK pathway; therefore, JAK signals serve as a key element in AD treatment. These cytokines can also directly activate sensory nerves with receptors, which promotes the pruritus sensation (20). The identification of the IL-4 receptor subunit *α* (IL-4Rα) on afferent neurons further emphasizes the interconnectedness between type 2 responses and neural itch. IL-31 can initiate itching by directly stimulating IL-31 receptor subunit α (IL-31Rα) and neurons that express TRPV1 or TRPA1 (1). As research progressed, relevant diagnosis and treatment guidelines gradually emerged. Advanced drugs have increasingly appeared, including crisaborole, dupilumab, upadacitinib, and nemolizumab; all of these drugs could improve the efficacy of AD treatment. Therefore, the application of advanced therapy was the most recent hot topic.

Research on crisaborole from 2010 to 2016 was highly popular; however, its popularity decreased sharply once it was released into the market (Figure 6C). Crisaborole ointment, a novel PDE4 inhibitor, could relieve pruritus, which is significant when treating patients with AD (21). Prurigo nodularis (PN) is a relatively rare dermatosis with keratotic nodules; it is distinguished by persistent chronic itching. Recent studies have partly unveiled the complex interaction between PN-associated itch and cutaneous neuroimmune phenomena (22). However, dupilumab has a certain effect on PN. Dupilumab, an antibody that blocks IL-4 and IL-13, can improve clinical responses in AD without significant safety concerns (10). Upadacitinib, which has gradually gained more attention since 2015, is a selective JAK1 inhibitor. In clinical trials, upadacitinib has shown efficacy in treating moderate-to-severe AD, particularly in those who display inadequate response to dupilumab or baricitinib (23). “Protein-coupled receptor” and “jak inhibitor” were hot keywords that appeared in 2020 (Figure 7D). In recent years, it was found that skin mast cells express Mas-related G-protein-coupled receptor X2 (MRGPRX2), which is a crucial protein involved in non-IgE-mediated MC degranulation, and its hyperactivity can contribute to the development of AD (24). JAK inhibitors can treat AD as a promising therapeutic agent by blocking cytokine, growth factor, and hormone receptor signaling pathways. These two keywords remain highly relevant until the recent search year.

Through bibliometric and visual analyses of the literature, we can gain deeper insights into trends and hot topics regarding AD-related itch. Nevertheless, this study possesses certain limitations. First, the data were confined to English-language articles and reviews sourced from SCI-E. Second, even though WoSCC is a high-quality database, its exclusive use may introduce selection bias into the analysis and thus limit the generalizability of findings. Third, bibliometric analysis is susceptible to time-effect biases. Fourth, whether the increase in publications reflects genuine scientific advancements or merely an increase in bibliometric output remains unclear due to the characteristics of bibliometrics. Finally, citation counts do not necessarily reflect research quality or clinical impact, and some controversial challenges involving AD-related itch need to be confirmed clinically in future studies.

## 5 Conclusion

The study represents the first bibliometric and visual analyses of research pertaining to AD-related itch. In the past decade, the growth of publications reflects the heightened interest in this research area. Earlier research was focused on exploring the mechanisms of AD-related itch. In recent years, advanced drugs have attracted extensive attention, including crisaborole, upadacitinib, dupilumab, and JAK inhibitors, and will be noteworthy research directions in the future. We anticipate that the study will provide scholars with a comprehensive understanding of the current situation of the research on AD-related itch, offering a broader perspective.

## Data Availability

The original contributions presented in the study are included in the article/Supplementary material, further inquiries can be directed to the corresponding authors.

## Footnotes

^1^ http://www.bioinformatics.com.cn/
