# Supplementary material for: Emerging trends and research hotspots in atopic dermatitis-related itch over the past 10 years: a bibliometric and visual analysis
Source: Front Med (Lausanne). 2025 Jun 17;12:1503312. doi: 10.3389/fmed.2025.1503312 (PMC12209377; doi:10.3389/fmed.2025.1503312)
Supplement: Supplementary file 1 [file Table_1.docx]

Supplementary Table 1. TS search queries and refinement procedure

| Set | Results | Refinement |
| --- | --- | --- |
| 1 | 4555 | TOPICS: (TS=("atopic dermatitis") OR TS= ("atopic eczema")) AND (TS=(itch) OR TS=(Pruritus)) |
| 2 | 2916 | Refined by PUBLICATION YEARS:(2014 OR 2015 OR 2016 OR 2017OR 2018 OR 2019 OR 2020 OR 2021 0R 2022 OR 2023) |
| 3 | 2613 | Refined by DOCUMENT TYPES: (ARTICLES OR REVIEW ARTICLES) |
| 4 | 2534 | Refined by LANGUAGES: (ENGLISH) |

Supplementary Table 2. The top ten countries with the highest productivity

| Rank | Country | NP | NC | H-index | Average citation per item |
| --- | --- | --- | --- | --- | --- |
| 1 | USA | 924 | 29248 | 88 | 36.74 |
| 2 | GERMANY | 381 | 17161 | 60 | 48.6 |
| 3 | JAPAN | 336 | 10510 | 54 | 34.12 |
| 4 | CHINA | 321 | 3933 | 31 | 13.19 |
| 5 | SOUTH KOREA | 225 | 2788 | 25 | 12.96 |
| 6 | ENGLAND | 177 | 7477 | 39 | 44.19 |
| 7 | FRANCE | 169 | 7495 | 39 | 46.22 |
| 8 | ITALY | 158 | 3311 | 30 | 22.1 |
| 9 | CANADA | 137 | 6127 | 35 | 46.23 |
| 10 | POLAND | 122 | 3698 | 25 | 31.29 |

Supplementary Table 3. The top ten most productive affiliations

| Rank | Affiliations | Country | NP | NC | | H-index | Average citation per item |
| --- | --- | --- | --- | --- | --- | --- | --- |
| 1 | NORTHWESTERN UNIVERSITY | USA | 148 | 3465 | 26 | | 46.92 |
| 2 | FEINBERG SCHOOL OF MEDICINE | USA | 140 | 16296 | 50 | | 275.37 |
| 3 | GEORGE WASHINGTON UNIVERSITY | USA | 112 | 7059 | 41 | | 51.43 |
| 4 | UNIVERSITY OF CALIFORNIA SYSTEM | USA | 108 | 6671 | 40 | | 51.28 |
| 5 | OREGON HEALTH SCIENCE UNIVERSITY | USA | 102 | 1599 | 18 | | 15.86 |
| 6 | ICAHN SCHOOL OF MEDICINE AT MOUNT SINAI | USA | 90 | 4329 | 33 | | 41.55 |
| 7 | UNIVERSITY OF MIAMI | USA | 87 | 7530 | 39 | | 76.92 |
| 8 | UNIVERSITY OF MUNICH | Germany | 71 | 6383 | 33 | | 73.3 |
| 9 | UNIVERSITY OF MUNSTER | Germany | 69 | 2010 | 23 | | 25.52 |
| 10 | UNIVERSITY OF COPENHAGEN | Denmark | 65 | 5708 | 27 | | 82.35 |

Supplementary Table 4. The top ten authors with the most publications

| Rank | Author | NP | NC | Country/Region | Affiliation | H-index | Average citation per item |
| --- | --- | --- | --- | --- | --- | --- | --- |
| 1 | Simpson EL | 92 | 6738 | USA | Oregon Health & Science University | 37 | 76.46 |
| 2 | Silverberg JI | 76 | 4606 | USA | Northwestern University | 31 | 65.11 |
| 3 | Yosipovitch G | 66 | 1460 | USA | University of Miami | 20 | 23.45 |
| 4 | Ständer S | 56 | 1192 | Germany | University of Munster | 21 | 22.32 |
| 5 | Guttman-Yassky E | 52 | 5492 | USA | Icahn School of Medicine at Mount Sinai | 30 | 108.33 |
| 6 | Wollenberg A | 45 | 5068 | Germany | University of Munich | 24 | 115.13 |
| 7 | Kabashima K | 40 | 3158 | Japan | Kyoto University | 23 | 81.25 |
| 8 | Paller AS | 40 | 2781 | USA | Northwestern University | 23 | 70.9 |
| 9 | Thyssen JP | 38 | 1365 | Denmark | University of Copenhagen | 17 | 36.95 |
| 10 | Szepietowsk JC | 36 | 1429 | Poland | Wroclaw Medical University | 15 | 40.86 |

Supplementary Table 5. The top ten most-published journals

| Rank | Journal | NP | NC | IF（2022） | H-index | Average citation per item |
| --- | --- | --- | --- | --- | --- | --- |
| 1 | ACTA DERMATO VENEREOLOGICA | 93 | 1664 | 3.6 | 24 | 18.52 |
| 2 | VETERINARY DERMATOLOGY | 90 | 984 | 1.4 | 17 | 12.72 |
| 3 | JOURNAL OF THE EUROPEAN ACADEMY OF DERMATOLOGY AND VENEREOLOGY | 88 | 2273 | 9.2 | 24 | 26.45 |
| 4 | INTERNATIONAL JOURNAL OF MOLECULAR SCIENCES | 69 | 970 | 5.6 | 17 | 14.38 |
| 5 | BRITISH JOURNAL OF DERMATOLOGY | 63 | 3325 | 10.3 | 31 | 53.65 |
| 6 | JOURNAL OF THE AMERICAN ACADEMY OF DERMATOLOGY | 63 | 3961 | 13.8 | 32 | 63.6 |
| 7 | DERMATOLOGY AND THERAPY | 57 | 392 | 3.4 | 12 | 7.11 |
| 8 | EXPERIMENTAL DERMATOLOGY | 55 | 918 | 3.6 | 17 | 17 |
| 9 | JOURNAL OF ALLERGY AND CLINICAL IMMUNOLOGY | 55 | 3975 | 14.2 | 31 | 73.93 |
| 10 | JOURNAL OF DERMATOLOGICAL TREATMENT | 55 | 662 | 2.9 | 14 | 12.22 |
